# Supplementing Synbiotic in Sows' Diets Modifies Beneficially Blood Parameters and Colonic Microbiota Composition and Metabolic Activity in Suckling Piglets

**DOI:** 10.3389/fvets.2020.575685

**Published:** 2020-11-30

**Authors:** Cui Ma, Qiankun Gao, Wanghong Zhang, Qian Zhu, Wu Tang, Francois Blachier, Hao Ding, Xiangfeng Kong

**Affiliations:** ^1^CAS Key Laboratory of Agro-Ecological Processes in Subtropical Region, Hunan Provincial Key Laboratory of Animal Nutritional Physiology and Metabolic Process, National Engineering Laboratory for Pollution Control and Waste Utilization in Livestock and Poultry Production, Institute of Subtropical Agriculture, Chinese Academy of Sciences, Changsha, China; ^2^College of Advanced Agricultural Sciences, University of Chinese Academy of Sciences, Beijing, China; ^3^Université Paris-Saclay, AgroParisTech, INRAE, UMR PNCA, Paris, France

**Keywords:** biochemical parameters, gut microbiota, metabolites, sows, suckling piglets, synbiotic

## Abstract

Nutrients in the maternal diet favor the growth and development of suckling piglets and alter their gut microbiota composition and metabolic activity, thus affecting the hosts. The present study analyzed, in suckling piglets from sows receiving antibiotic or synbiotic supplements from pregnancy to lactation, several biochemical parameters, oxidative/anti-oxidative indices, inflammatory cytokines, and ingestion-related factor levels in plasma, as well as colonic microbiota composition and metabolic activity, and mucosal expression of genes related to the intestinal barrier function. Compared with the control group, maternal synbiotic supplementation decreased (*P* < 0.05) the plasma levels of glucose, AMM, TC, low-density lipoprotein-cholesterol (LDL-C), MDA, H_2_O_2_, ghrelin, CCK, PP, IL-1β, IL-2, IL-6, TNF-α, Ala, Cys, Tau, and β-AiBA, the levels of propionate and total short-chain fatty acids (SCFAs) in the colonic luminal content, and colonic abundances of *RFN20, Anaerostipes*, and *Butyricimonas*; while increased (*P* < 0.05) the plasma levels of urea nitrogen (UN), Ile, Leu, α-AAA, α-ABA, and 1-Mehis, as well as colonic abundances of *Sphingomonas, Anaerovorax, Sharpea*, and *Butyricicoccus*. Compared with the antibiotic group, maternal synbiotic supplementation decreased (*P* < 0.05) the plasma levels of glucose, gastrin, and Ala, as well as abundances of *Pasteurella* and *RFN20* and propionate level in the colonic content. Expression of genes coding for E-cadherin, Occludin, ZO-1, ZO-2, IL-10, and interferon-α were down-regulated in the colonic mucosa. The synbiotic supplementation increased (*P* < 0.05) the plasma levels of UN, Leu, α-ABA, and 1-Mehis, the abundances of *Anaerovorax, Sharpea*, and *Butyricicoccus* and expression of genes coding for E-cadherin, Occludin, ZO-1, ZO-2, IL-10, and interferon-α. Spearman correlation analysis showed that there was a positive correlation between colonic *Anaerostipes* abundance and acetate and SCFAs levels; whereas a negative correlation between *Fusobacteria* and *Fusobacterium* abundances and acetate level. These findings suggest that synbiotic supplementation in the maternal diet improved nutrient metabolism and intestinal barrier permeability, reduced oxidative stress, and modified colonic microbiota composition and metabolic activity in suckling piglets.

## Introduction

Economic benefit in swine farm is directly affected by the survival rate, growth and development, and health of suckling piglets (1). The survival and health of suckling piglets are largely dependent on maternal milk quality (2). Maternal nutrition during lactation is an important factor affecting the quality and quantity of the maternal milk. Therefore, improving maternal nutrient level could help to enhance sows lactating performance and promote the growth and development of piglets.

Gut microbiota is involved in the metabolism, growth, and development of the host (3). Short-chain fatty acids (SCFAs) are products of some specific gut bacteria and could serve as luminal energy substrates in colonocytes (4). In addition, SCFAs exert an anti-inflammatory effect in the gut (5). Microbiota colonization in infant gut begins from their mother's wombs (6) and is affected by diets and other environmental factors (7). Exposure to antibiotics *via* oral administration as a kind medicine (especially the broad-spectrum antibiotics) in newborn animals has a major effect on gut microbiota composition (8). Antibiotics was reported to promote nutrient absorption and increase the piglet growth (9). However, antibiotic overuse leads to drug residues in animals and their products, thus leading to antibiotic resistance and affecting humans health (10). Synbiotics, the mixed additive of prebiotics and probiotics, have shown several beneficial effects in pig production. For instance, several studies showed that dietary synbiotic supplementation improved the intestinal microbiota and growth performance of weaned piglets (11, 12). Therefore, we speculated that synbiotics in the maternal diet could affect the offspring, notably by modifying the gut microbiota and metabolic activity.

Our previous study showed that dietary synbiotic supplementation increased the piglet survival rate by improving the glycolipids absorption and utilization and altering the gut microbiota composition and abundances of sows (13). The present study hypothesizes that maternal synbiotic supplementation may modify beneficially blood indices, gut microbiota composition and metabolic activity, and the mucosal mRNA expression of genes related to the intestinal barrier function. Therefore, the effects of synbiotic supplementation in sows' diets were measured on several parameters in suckling piglets, including plasma biochemical parameters, oxidative/anti-oxidative indices, inflammatory and ingestion-related factors, and free amino acids. In addition, colonic microbiota composition and metabolic activity were measured in piglets, as well as expression of colonic mucosa genes involved in epithelial barrier function and inflammation.

## Materials and Methods

### Experimental Design

The animal experiment was conducted in Hantang Agriculture Co. Ltd., Shimen, Hunan, China. Forty-eight pregnant Bama mini-pigs were selected and randomly allocated into one of three groups (16 sows per group). The sows in the control group were fed a basal diet, those in the antibiotic group were fed a basal diet supplemented with 50 g/t virginiamycin, and those in the synbiotic group were fed a basal diet supplemented with 200 mL/d fermentation broth per animal and 500 g xylo-oligosaccharides (XOS) per ton diet. The fermentation broth was provided by Hunan Lifeng Biotechnology Co. Ltd. and contained ≥ 1.2 × 10^8^ CFU/g viable *Lactobacillus plantarum* B90 (BNCC1.12934) ≥ 1.0 × 10^8^ CFU/g and *Saccharomyces cerevisiae* P11 (BNCC2.3854) ≥ 0.2 × 10^8^ CFU/g. The XOS was provided by Shandong Longlive Biotechnology Co., Ltd., Shandong, China; and contained xylobiose, xylotriose, and xylotetraose at level ≥ 35%. The diet composition and nutrient levels for the sows met the Chinese pig local standard (NY-2004), and the premixes for pregnant and lactating sows met the NRC recommended requirements (NRC, 2012) (Supplementary Table 1). The experimental period was from mating to weaning (postpartum 21 d). During the trial period, there were four sows returned to estrus in the control group, two sows returned to estrus in the antibiotic group, and three sows returned to estrus in the synbiotic group. The diets were fed twice daily (8:00 a.m. and 5:00 p.m.) fluctuating with the physical condition of the sows throughout the trail, and water was available freely.

### Sample Collection and Preparation

At 21 day-old (weaned), the piglets from 12 litters were weighed after fasted for about 12 h and one piglet with middle body weight (BW) per litter was selected. Twelve piglets per group were exsanguinated after electrical stunning (120 V, 200 Hz). Each piglet per group was randomly chosen to collect blood samples from precaval vein into 10 mL heparin coated-tubes and plasma was separated by centrifuging at 3,500 g and 4°C for 10 min and stored at −20°C for further analysis. Colonic contents (middle section) were collected in 10 mL sterile centrifuge tubes and stored immediately at −20°C for subsequent analysis of microbiota composition and metabolites. After washing with cold physiological saline, the colonic mucosal tissues were sampled and immediately frozen in liquid nitrogen (~2 g), and then stored at −80°C for mRNA analyses.

### Determination of Plasma Biochemical Parameters

The plasma levels of albumin (ALB), alkaline phosphatase (ALP), alanine aminotransferase (ALT), ammonia (AMM), aspartate aminotransferase (AST), glucose (GLU), high-density lipoprotein-cholesterol (HDL-C), low-density lipoprotein-cholesterol (LDL-C), total cholesterol (TC), triglyceride (TG), total protein (TP), and urea nitrogen (UN) were determined using commercially available kits (F. Hoffmann-La Roche Ltd, Basel, Switzerland) with the Roche automatic biochemical analyzer (Cobas c311, F. Hoffmann-La Roche Ltd, Basel, Switzerland).

### Determination of Plasma Oxidative/Anti-oxidative Indices, Inflammatory Cytokines, and Ingestion Related Factors

The plasma levels of catalase (CAT), hydrogen peroxide (H_2_O_2_), malondialdehyde (MDA), superoxide dismutase (SOD), and total antioxidant capacity (T-AOC), were determined as per commercially available kit directions (Suzhou keming, Co. Ltd, Jiangsu, China) with Multiscan Spectrum (Tecan, Infinite M200 Pro, Switzerland).

The plasma levels of gastrin, ghrelin, cholecystokinin (CCK), interleukin (IL)-1β, IL-2, IL-6, IL-10, interferon (IFN)-α, insulin-like growth factor (IGF)-1, leptin (LEP), pancreatic polypeptide (PP), peptide YY (PPY), and tumor necrosis factor (TNF)-α were measured according to the Meimian ELISA kit directions (Jiangsu Yutong Biological Technology, Co. Ltd., Jiangsu, China) on Multiscan Spectrum (Tecan, Infinite M200 Pro, Switzerland).

### Determination of Plasma Free Amino Acids

Approximately 1.00 mL plasma sample was added into 1.00 mL 8% salicylic acid solution, mixed thoroughly and overnighted at 4°C, and then centrifuged at 8,000 r/min for 10 min to obtain the supernatant. The processed samples were filtered through a 0.45-μm membrane prior to analysis of free amino acids with an automatic AA analyzer (L8900, Hitachi, Tokyo, Japan).

### DNA Extraction and 16S rRNA Gene Sequencing

The total genomic DNA of colonic content samples was extracted using the Fast DNA SPIN extraction kits (MP Biomedicals, Santa Ana, CA, USA). The DNA concentration was determined using NanoDrop ND-1000 spectrophotometer (Thermo Fisher Scientific, Waltham, MA, USA). The V3-V4 regions was amplified using the primer 338F (5′-GCACCTAAYTGGGYDTAAAGNG-3′) and 806R (5′-TACNVGGGTATCTAATCC-3′). The protocol of PCR amplification was conducted according to our previous study (13). The PCR products were successfully separated using 1.2% agarose gel electrophoresis, purified using Agencourt AMPure Beads (Beckman Coulter, Indianapolis, IN), and further quantified using the PicoGreen dsDNA Assay Kit (Invitrogen, Carlsbad, CA, USA) according to the manufacturer's instructions. Purified amplicons were then subjected to paired-end (2 × 300) sequencing on an Illumina MiSeq platform (Illumina, San Diego, USA) using the MiSeq Reagent Kit v3 (600 cycles) according to the standard protocol, which was performed by Shanghai Personal Biotechnology Co. Ltd., Shanghai, China. The raw Illumina pair-end read data for all samples are available in the NCBI Sequence Read Archive with accession number PRJNA609410.

### Determination of Metabolites in Colonic Contents

The SCFAs in colonic contents were measured with gas chromatography (Agilent Technologies 1206, Santa Clara, CA, USA) according to the previous description (14). The levels of bioamines, indole, and skatole in colonic contents were measured using reverse phase-high performance liquid chromatography (Agilent Technologies, Santa Clara, CA, USA) according to a previous study (14).

### Determination of mRNA Expression of Genes Related to Intestinal Health

The primers for target genes and reference gene β-actin (listed in Supplementary Table 2) were designed using Primer-BLAST. RNA extraction and real-time polymerase chain reaction (RT-PCR) analyses were conducted as a previous report (15). The relative expression level of each target gene was determined by RT-PCR with performing on a 480II system (Roche, LightCycler® 480II, Switzerland) and calculated by the 2^−ΔΔCt^ method (16).

### Statistical Analysis

The plasma indices, colonic metabolite levels, and colonic microbiota alpha diversity were analyzed using one-way analysis of variance (ANOVA) followed by Duncan's multiple range *post hoc* test with SPSS 22. The microbial community structural variation among samples was performed by the beta diversity analysis (PERMANOVA) (17) and was showed using the partial least squares-discriminant analysis (PLS-DA). The colonic microbiota abundance and overall composition at phyla and genus levels were analyzed using Metastats (http://metastats.cbcb.umd.edu/) (18). The graph preparation was performed using GraphPad Prism ver7.0 (San Diego, CA, USA). Spearman's correlation between colonic microbiota abundances and metabolite levels was analyzed with the R package. All data were presented as means ± SEM. Differences were considered statistically significant at *P* < 0.05.

## Results

### Plasma Biochemical Parameters of Piglets

**Figure 1:**
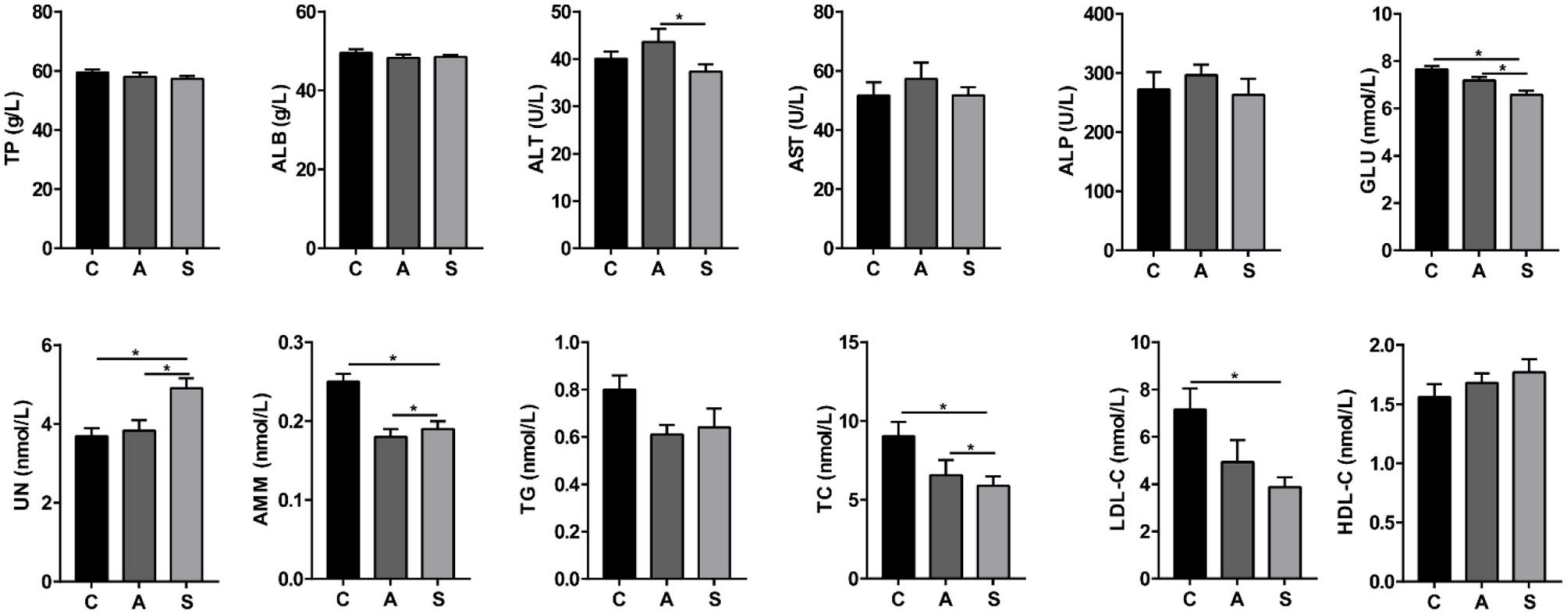
Effect of maternal synbiotic supplementation on plasma biochemical parameters of suckling Bama mini-piglets. C, A, and S present the control group, antibiotic group, and synbiotic group, respectively. The same as below. Data represent the means ± SEM. *indicates statistically significant (*P* < 0.05). *n* = 8 per group.

As shown in Figure 1, compared with the control group, maternal synbiotic supplementation increased (*P* < 0.05) plasma UN level while decreased (*P* < 0.05) plasma GLU, AMM, TC, and LDL-C levels. Maternal synbiotic supplementation decreased (*P* < 0.05) plasma ALT and GLU levels, increased (*P* < 0.05) UN level, and showed an increased trend in TG level (*P* = 0.074), when compared with the antibiotic group.

### Plasma Oxidative/Anti-oxidative Indices, Inflammatory Cytokines, and Ingestion Related Factors of Piglets

**Figure 2:**
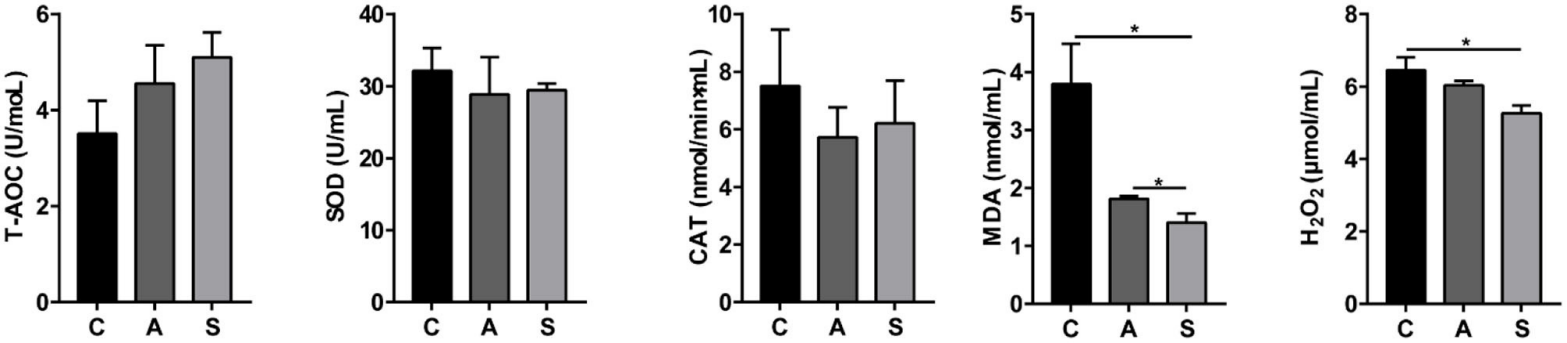
Effect of maternal synbiotic supplementation on plasma oxidative/anti-oxidative levels in suckling Bama mini-piglets. Data represent the means ± SEM. *indicates statistically significant (*P* < 0.05). *n* = 8 per group.

As shown in Figure 2, compared to the control group, maternal synbiotic supplementation decreased (*P* < 0.05) plasma MDA and H_2_O_2_ levels and antibiotic supplementation decreased (*P* < 0.05) plasma MDA level. However, the plasma T-AOC, SOD, and CAT indices did not reach statistical significance (*P* > 0.05).

**Figure 3:**
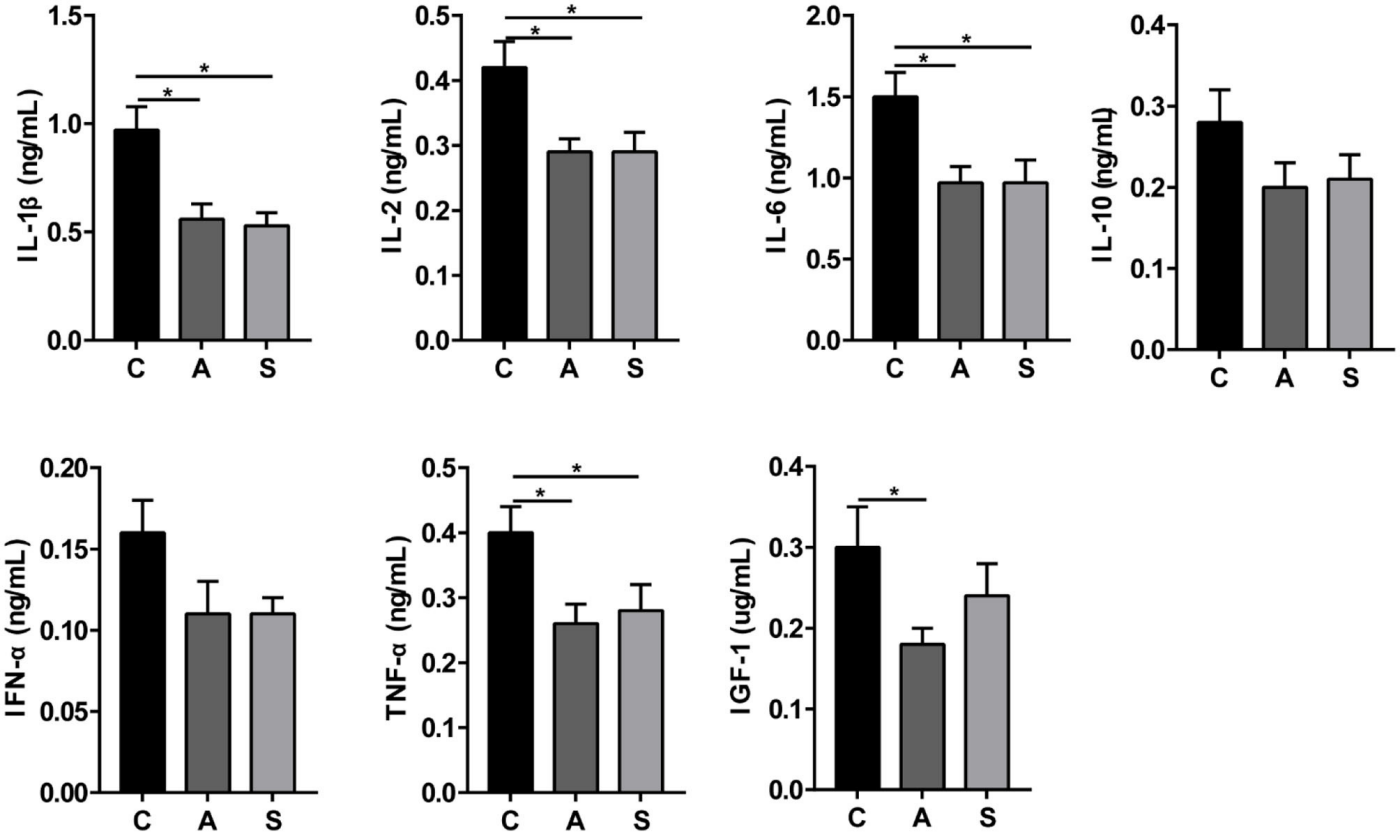
Effect of maternal synbiotic supplementation on plasma inflammatory cytokine levels in suckling Bama mini-piglets. Data represent the means ± SEM. *indicates statistically significant (*P* < 0.05). *n* = 8 per group.

As presented in Figure 3, maternal synbiotic supplementation decreased (*P* < 0.05) plasma levels of IL-1β, IL-2, IL-6, and TNF-α; and antibiotic supplementation decreased (*P* < 0.05) plasma levels of IGF-1, IL-1β, IL-2, IL-6, and TNF-α, when compared with the control group.

**Figure 4:**
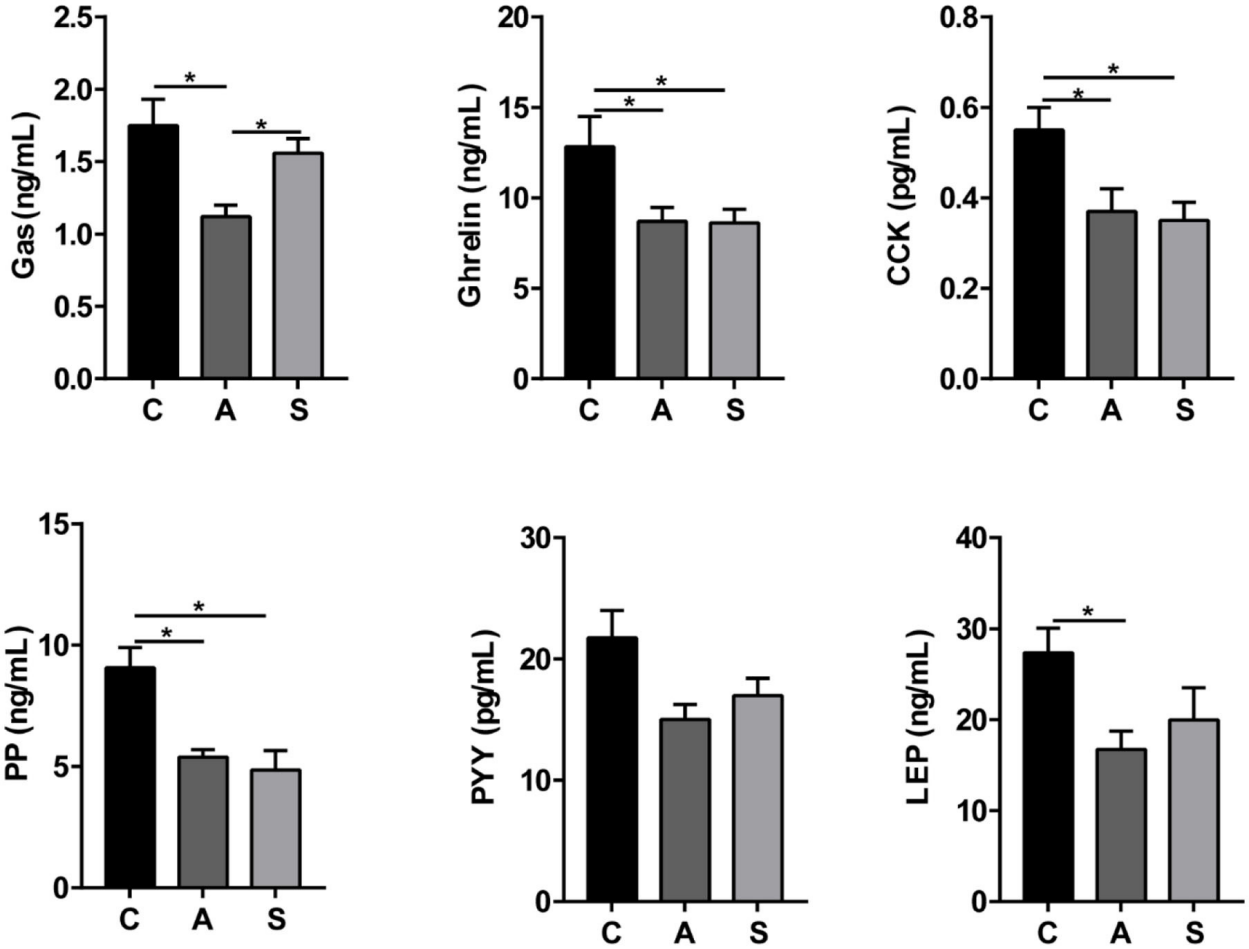
Effect of maternal synbiotic supplementation on plasma ingestion-related factor levels in suckling Bama mini-piglets. Data represent the means ± SEM. ^*^indicates statistically significant (*P* < 0.05). *n* = 8 per group.

As listed in Figure 4, maternal synbiotic supplementation decreased (*P* < 0.05) plasma ghrelin, CCK, and PP levels and had a decreased trend in LEP level (*P* = 0.05); and maternal antibiotic supplementation decreased (*P* < 0.05) plasma gastrin, ghrelin, CCK, PP, LEP, and SS levels, when compared with the control group. Maternal synbiotic supplementation decreased plasma gastrin (*P* < 0.05) and LEP (*P* = 0.05) levels relative to the antibiotic group.

### Plasma Free Amino Acid Levels of Piglets

As shown in Table 1, maternal synbiotic supplementation decreased (*P* < 0.05) plasma Ile, Leu, α-AAA, α-ABA, and 1-Mehis levels and antibiotic supplementation decreased (*P* < 0.05) plasma Hypro level, when compared with the control group. The plasma Leu, α-ABA, and 1-Mehis levels in the synbiotic group was higher (*P* < 0.05) while plasma Ala level was lower (*P* < 0.05) compared with the antibiotic group.

**Table 1:** Effects of maternal synbiotic supplementation on plasma concentrations of free amino acids in suckling Bama mini-piglets (μg/mL; *n* = 8).

**Items**	**Control group**	**Antibiotic group**	**Synbiotic group**
Ala	29.58 ± 2.90^a^	28.04 ± 2.83^a^	13.64 ± 1.5^b^
Ans	0.87 ± 0.17	0.51 ± 0.03	0.57 ± 0.08
Arg	18.44 ± 1.55	16.06 ± 0.56	18.60 ± 1.60
Asp	2.45 ± 0.54	2.48 ± 0.24	1.96 ± 0.21
Car	7.18 ± 0.36	8.37 ± 0.72	5.86 ± 0.44
Cit	9.28 ± 0.58	8.93 ± 0.66	10.91 ± 0.83
Cys	0.85 ± 0.15^a^	1.69 ± 0.37^ab^	1.95 ± 0.21^b^
Cysthi	3.43 ± 0.33	3.31 ± 0.13	4.12 ± 0.31
EOHNH_2_	0.56 ± 0.39	1.29 ± 0.56	3.10 ± 0.24
Glu	39.12 ± 9.02	27.93 ± 3.12	21.61 ± 1.97
Gly	31.35 ± 1.92	35.56 ± 2.24	31.80 ± 1.06
His	10.27 ± 0.52	10.74 ± 0.55	11.10 ± 0.69
Hypro	10.52 ± 0.85^b^	12.88 ± 0.57^a^	12.08 ± 0.7^ab^
Ile	15.73 ± 1.98^b^	16.44 ± 1.5^ab^	21.23 ± 1.53^a^
Leu	19.66 ± 1.79^b^	20.76 ± 1.96^b^	26.94 ± 1.92^a^
Lys	20.25 ± 1.69	22.22 ± 2.17	23.60 ± 0.79
Met	3.90 ± 0.46	3.80 ± 0.37	3.97 ± 0.21
Orn	6.76 ± 0.61	7.32 ± 0.65	6.62 ± 0.44
Phe	13.88 ± 0.93	14.68 ± 0.64	15.58 ± 0.40
Pro	16.35 ± 1.14	16.48 ± 1.10	18.37 ± 0.88
Sar	1.09 ± 0.23	1.42 ± 0.43	1.30 ± 0.33
Ser	11.92 ± 1.02	12.47 ± 1.06	10.96 ± 0.82
Tau	9.97 ± 0.36^a^	9.42 ± 0.75^ab^	8.16 ± 0.42^b^
Thr	17.31 ± 1.10	16.7 ± 1.21	15.56 ± 1.20
Tyr	11.31 ± 1.38	11.28 ± 1.00	10.66 ± 0.53
Val	32.45 ± 4.11	32.73 ± 3.56	37.71 ± 2.38
α-AAA	6.86 ± 0.84^b^	7.86 ± 0.93^ab^	9.80 ± 0.73^a^
α-ABA	3.55 ± 0.39^b^	3.43 ± 0.51^b^	4.94 ± 0.32^a^
β-AiBA	0.25 ± 0.04^a^	0.18 ± 0.02^ab^	0.40 ± 0.16^b^
β-Ala	1.00 ± 0.12	1.11 ± 0.14	1.20 ± 0.22
1-Mehis	0.38 ± 0.04^b^	0.55 ± 0.11^b^	1.21 ± 0.22^a^
3-Mehis	2.29 ± 0.12	2.05 ± 0.12	2.23 ± 0.17

*Data in the same row with different superscripts differ significantly (P < 0.05). Asp: Asp + Asn; Glu: Glu + Gln; α-AAA, L-alpha-aminoadipic acid; α-ABA, DL-alpha-amino-n-butyric acid; β-AiBA, DL-beta-aminoisobutyric acid; β-Ala, beta-alanine; 1-Mehis, L-1-methylhistidine; 3-Mehis, L-3-methylhistidine*.

### Diversity of Colonic Microbiota in Piglets

**Figure 5:**
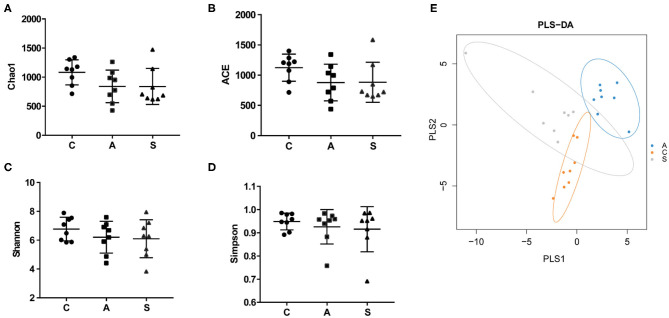
Effect of maternal synbiotic supplementation on alpha diversity of colonic microbiota in suckling Bama mini-piglets. **(A–D)** The microbial diversity is estimated by Chao, ACE, Shannon, and Simpson indices. **(E)** Partial least squares discrimination analysis (PLS-DA) of the colonic microbial community. Data represent the means ± SEM. The data were analyzed by One-way analysis of variance and Duncan's multiple range test. *n* = 8 per group.

Total 993,960 high-quality reads were generated from 48 colonic content samples, and each sample contained an average of 41,415 reads (range from 31,377 to 57,987). As shown in Figure 5, the Chao1, ACE, Simpson, and Shannon indices showed no difference among the three groups (*P* > 0.05). PLS-DA showed that samples from the three groups tended to exhibit a distinct clustering of microbiota composition although there was a partial overlap between the antibiotic group and synbiotic group.

### Composition and Abundance of Colonic Microbiota in Piglets

**Figure 6:**
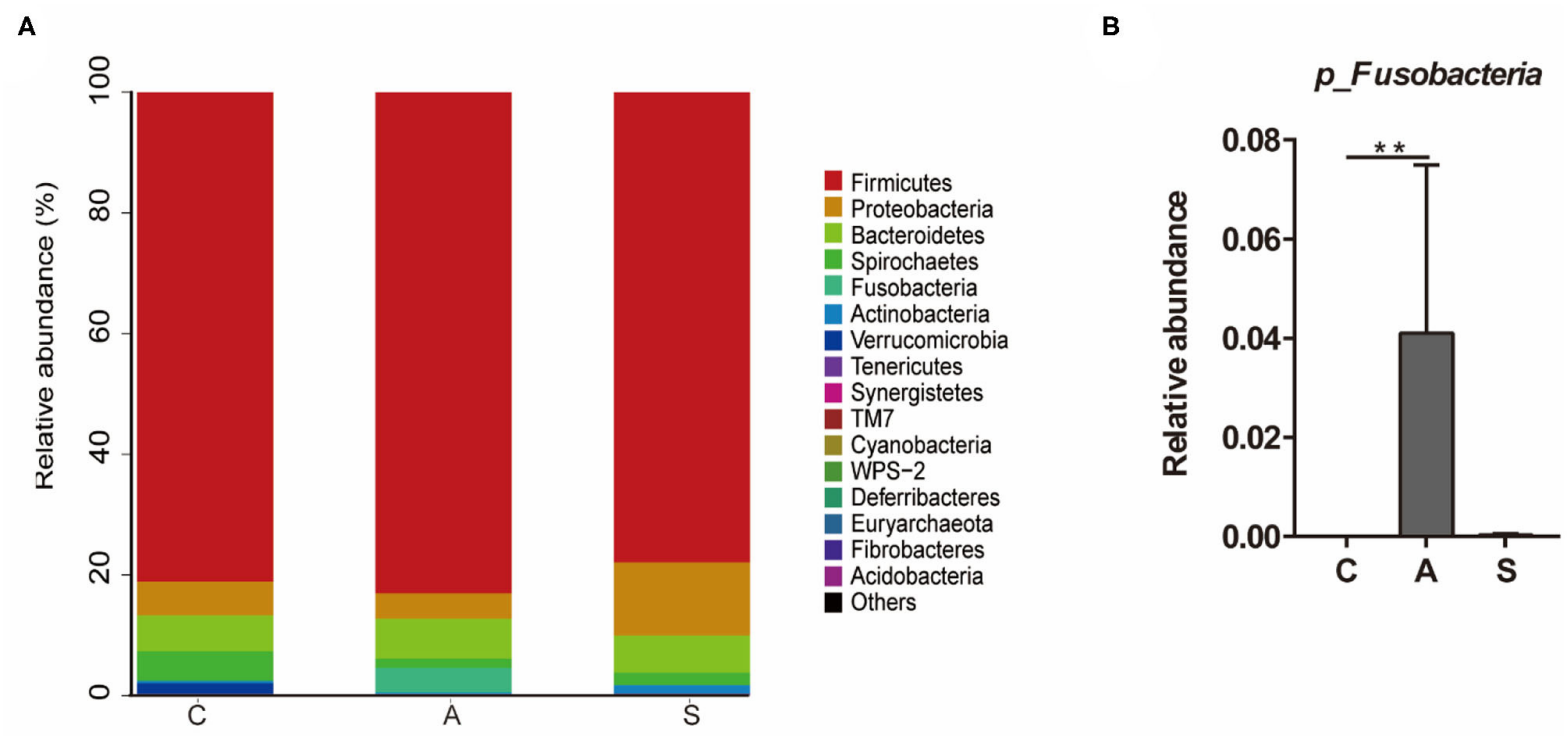
Effect of maternal synbiotic supplementation on the colonic microbial community structure in suckling Bama mini-piglets. Colonic microbiota distributed at the phylum level **(A)** and all phyla were listed. A comparison of relative abundances at the phylum level **(B)** was analyzed by Metastats analysis, and the discrepancy of the top 10 colonic microbiota was listed. Phyla with proportion < 0.001 were grouped in others. **P < 0.01. *n* = 8 per group.

As shown in Figure 6, the top five dominant phyla were *Firmicutes* (80.7%), *Proteobacteria* (7.3%), *Bacteroidetes* (6.3%), *Spirochaetes* (2.8%), and *Fusobacteria* (1.4%), which account for > 98% of total colonic bacteria. At phylum level, only *Fusobacteria* relative abundance in the antibiotic group was higher (*P* < 0.01) than that in the control group.

**Figure 7:**
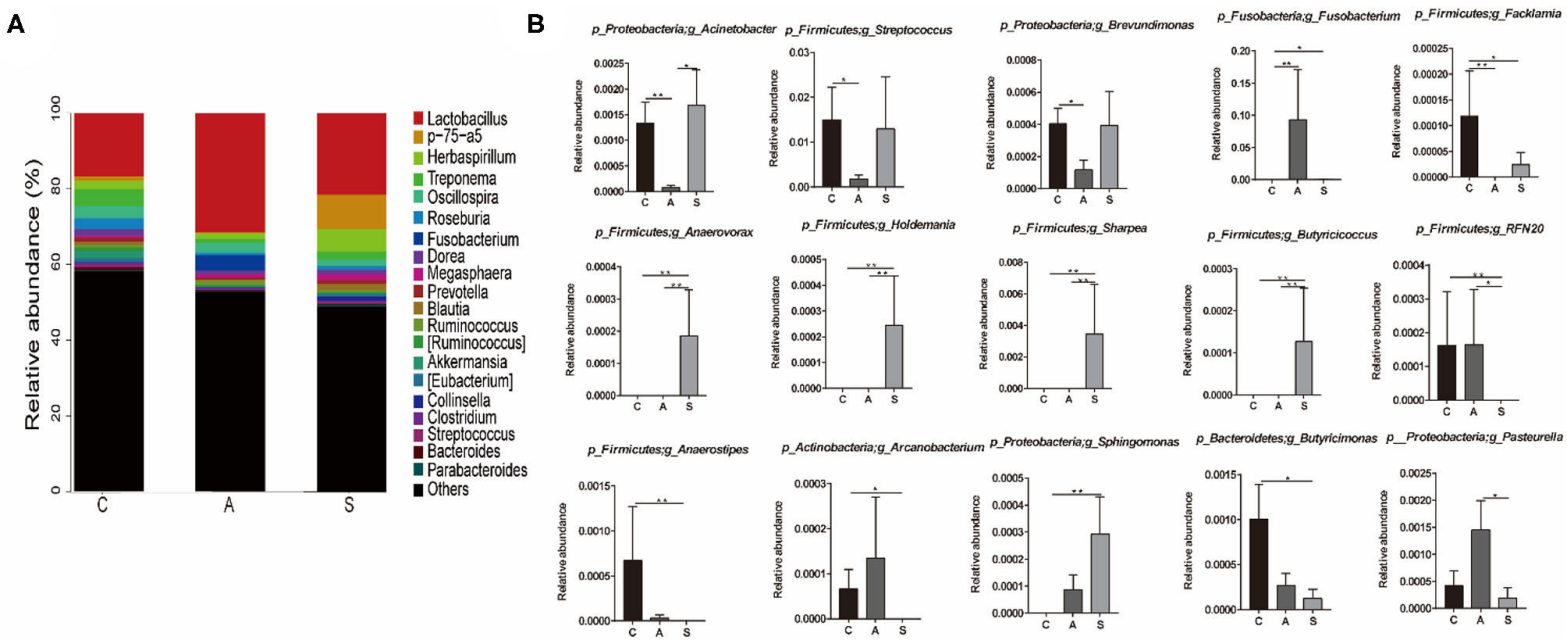
Effect of maternal synbiotic supplementation on the colonic microbial community structure in suckling Bama mini-piglets. Colonic microbiota distributed at the genus level **(A)** and only the top 20 genera were listed. A comparison of relative abundances at the genus level **(B)** was analyzed by Metastats analysis. The 20 most abundant bacterial genera with a clear classification status were presented and compared. *P < 0.05; **P < 0.01. *n* = 8 per group.

At genus level, *Lactobacillus* (23.2%)*, p-75-a5* (3.4%)*, Herbaspirillum* (3.3%), *Treponema* (2.5%), and *Oscillospira* (2.5%) were the top dominant genera of colonic microbiota with a clear classification status (Figure 7). Further, the abundances of colonic microbiota with a clear classification status of 20 most abundant bacterial genera were analyzed. Relative to the control group, maternal synbiotic supplementation increased (*P* < 0.05) the abundances of *p_Proteobacteria;g_Sphingomonas, p_Firmicutes;g_Anaerovorax, p_Firmicutes;g_Holdemania, p_Firmicutes;g_Sharpea, p_Firmicutes;g_Butyricicoccus*, and *p_Firmicutes;g_Anaerostipes*; while decreased (*P* < 0.05) the abundances of *p_Firmicutes;g_Facklamia, p_Firmicutes;g_RFN20, p_Actinobacteria;g_Arcanobacterium*, and *p_Proteobacteria;g_Brevundimonas*. Maternal antibiotic supplementation decreased (*P* < 0.05) the abundances of *p_Proteobacteria;g_Acinetobacter, p_Firmicutes;g_Facklamia, p_Firmicutes;g_Streptococcus*, and *p_Proteobacteria;g_Brevundimonas* while increased (*P* < 0.05) *p_Fusobacteria;g_Fusobacterium* abundance. Compared with the antibiotic group, maternal synbiotic supplementation decreased (*P* < 0.05) the abundances of *p_Proteobacteria;g_Pasteurella* and *p_Firmicutes;g_RFN20*, while increased (*P* < 0.01) the abundances of *p_Firmicutes;g_Anaerovorax, p_Firmicutes;g_Holdemania, p_Firmicutes;g_Sharpea*, and *p_Firmicutes;g_Butyricicoccus*.

### Metabolite Levels in Colonic Contents of Piglets

**Figure 8:**
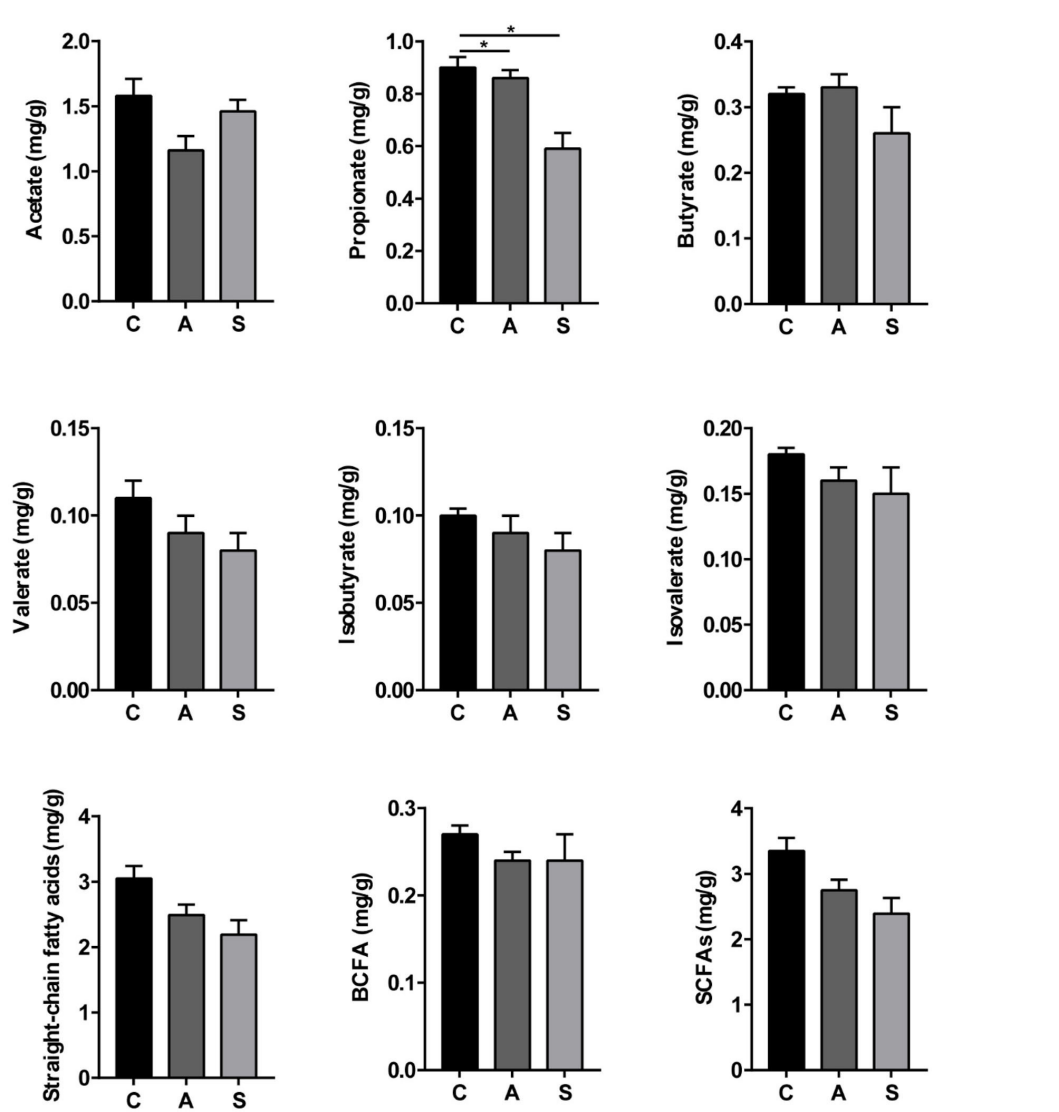
Effect of maternal synbiotic supplementation on colonic short-chain fatty acids levels of suckling Bama mini-piglets. The data were analyzed by Duncan's multiple range test using One-way analysis of variance. Data represent the means ± SEM. *P < 0.05. *n* = 8 per group.

As shown in Figure 8, compared with the control group, the levels of propionate, straight-chain fatty acids, and SCFAs were decreased (*P* < 0.05) and spermidine level showed a decreased trend (*P* = 0.055) in the synbiotic group. Moreover, maternal synbiotic supplementation decreased (*P* < 0.05) the propionate level and increased (*P* = 0.055) spermidine level compared with the antibiotic group. The differences in other determined metabolites among the three groups did not present statistically significant (*P* > 0.05) (Supplementary Figure 1).

### Correlation Between Microbiota and Metabolites in Colonic Content of Piglets

**Figure 9:**
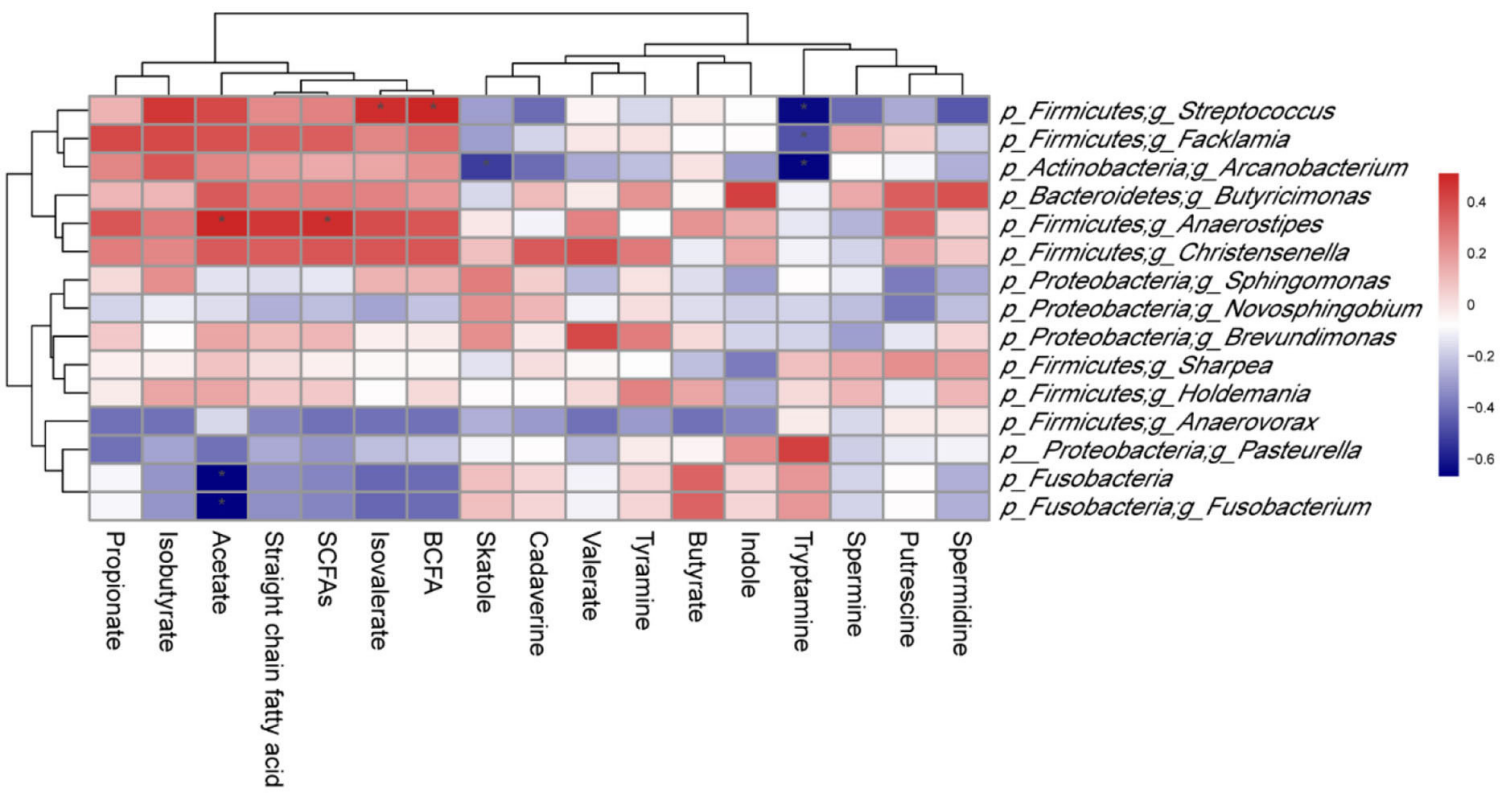
Correlation between colonic microbiota and their metabolites in suckling Bama mini-piglets. Spearman (r) correlations were used, and * means that the correlation is significant. SCFAs, short-chain fatty acids; BCFA, branched-chain fatty acid.

As shown in Figure 9, *p_Firmicutes;g_Butyricicoccus* abundance was positively correlated (*P* < 0.05) with isovalerate and branched-chain fatty acid (BCFA) levels, as well as *p_Firmicutes;g_Anaerostipes* abundance with acetate and SCFAs levels. However, a significant negative correlation (*P* < 0.05) was observed between *p_Fusobacteria* and *p_Fusobacteria;g_Fusobacterium* abundances and acetate level. In addition, there was a negative correlation (*P* < 0.05) between *p_Firmicutes;g_Facklamia* abundance and tryptamine level, as well as *p_Actinobacteria;g_Arcanobacterium* abundance and tryptamine and skatole levels.

### mRNA Expression of Genes Related to Intestinal Health in Piglets

**Figure 10:**
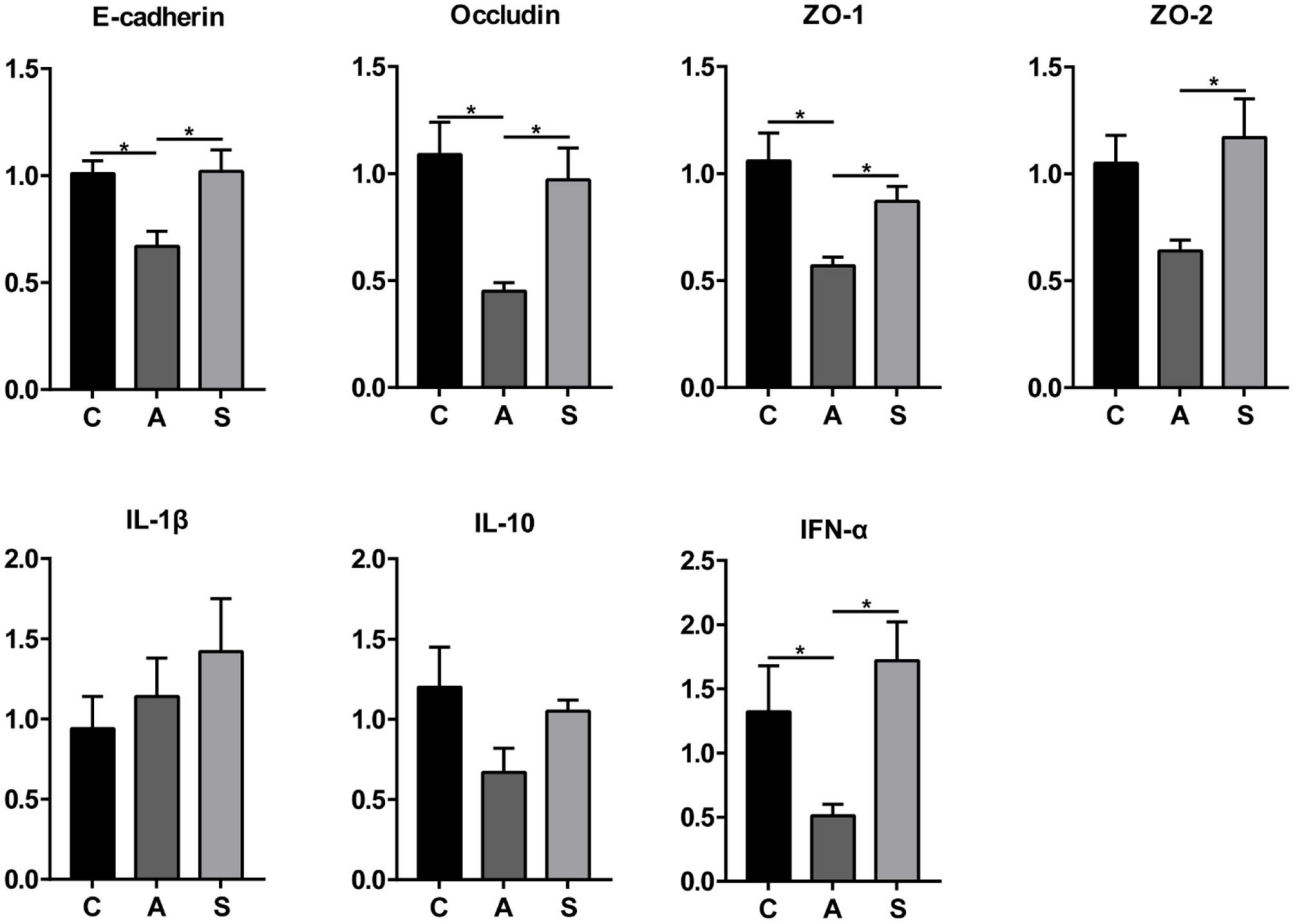
Effect of maternal synbiotic supplementation on mRNA expression of colonic mucosal genes related to the intestinal barrier function in suckling Bama mini-piglets. The data were analyzed by Duncan's multiple range test using One-way analysis of variance. Data showed the means ± SEM. *P < 0.05. *n* = 8 per group.

As shown in Figure 10, maternal synbiotic supplementation up-regulated (*P* < 0.05) the mRNA expression of colonic E-cadherin, Occludin, ZO-1, ZO-2, IL-10, and IFN-α compared with the antibiotic group. Compared with the control group, maternal synbiotic and antibiotic supplementation failed to affect the expression of determined genes.

## Discussion

The present study explored the effects of synbiotic supplementation in the maternal diets from pregnancy to lactation on the intestine health of suckling piglets by determining colonic microbiota composition, metabolite levels, and mucosal gene expression, as well as plasma parameters. We found that maternal antibiotic supplementation is counter-productive for the intestinal health based on the measurement of parameters related to the intestinal barrier permeability, whereas synbiotic supplementation improved parameters related to nutrient metabolism and intestinal health.

The piglets utilize efficiently dietary fat when blood TC level decreases. LDL-C transports TC synthesized by the liver to extrahepatic tissue, thus preventing excessive lipid deposition in the liver (19). In the present study, maternal synbiotic supplementation decreased plasma TC and LDL-C levels, suggesting that dietary fat was highly utilized by piglets to favor their growth. Shakeri et al. (20) reported that supplementing synbiotics reduced the blood TC level by altering gut microbiota metabolism. UN is a metabolite of amino acid and/or protein (21), plasma level of which reflects the profiles of protein absorption and utilization in the animal body (22). AMM reflects the liver function and the decrease of plasma AMM level indicates the increase of liver ability for synthesizing urea (23). The present study showed that plasma UN level increased while AMM level decreased in the synbiotic group, suggesting that maternal synbiotic supplementation promoted protein utilization of suckling piglets. These findings suggest that maternal synbiotic supplementation, but not antibiotic supplementation, would enhance the nitrogen metabolism of suckling piglets.

Amino acids (AAs), apart for being an important component of tissue protein, play several important roles in protein metabolism in animals (24). Weanling piglets use branched-chain amino acids (including Ile, Leu, and Val) to maintain their growth and development, especially Leu which contributes to regulate protein synthesis and tissue growth of animals (25). In the present study, maternal synbiotic supplementation increased the plasma Ile and Leu levels in suckling piglets. In addition, previous studies showed that Tau and Cys, main products of Met metabolism, play a vital role in the growth and health of piglets (26). Ala is the main substrate for glucose synthesis in the liver, which can play a role in the body's immune function (27). Tau, mostly found at a high level in animal tissues, has been shown to improve animal lipid metabolism (28). The present study showed that maternal synbiotic supplementation decreased the plasma levels of Tau, Cys, and Ala in piglets, suggesting that dietary synbiotics may modify amino acid metabolism in the offspring. These above-mentioned findings suggested that maternal synbiotic supplementation affects the protein synthesis by altering plasma amino acids levels.

Plasma MDA level reflects lipid peroxidation in the body tissues (29). H_2_O_2_ is a reactive oxygen species (ROS) that can increase the oxidative stress in tissues (30). A previous study showed that piglets may produce excessive reactive oxygen species thus leading to oxidative stress, which may lead to intestinal barrier dysfunction in weaned piglets (31). Interestingly, we found that maternal synbiotic supplementation decreased plasma MDA and H_2_O_2_ levels, suggesting that the synbiotics could relieve the oxidative stress exposure to suckling piglets. Among prebiotics, XOS produces SCFAs which may reduce ROS production (32), *Lactobacillus* reduces MDA production (33), and synbiotic addition reduces the MDA level and relieves oxidative stress in tissues (29).

Gut microbiota is involved in nutrient utilization and affects the growth and development of the host (34). Maternal nutrition during pregnancy and lactation modified the gut microbiota composition and health of offspring (35). Gut microbiota diversity was closely related with the host's health (36). The α-diversity of microbiota is decreased, which may be associated with a higher occurrence of low-grade inflammation and some metabolic diseases (37). In the present study, after maternal antibiotic or synbiotic supplementation, the α-diversity of colonic microbiota in piglets did not change, whereas the microbiota composition and abundances changed markedly, suggesting that maternal synbiotic might not exert a negative effect on suckling piglets.

In the animal gut, the dominant phyla usually includes *Firmicutes, Bacteroides, Proteobacteria*, and *Fusobacterium* (38). In the present study, the abundances of *Firmicutes, Bacteroides*, and *Proteobacteria* accounted for 94.3% of the total sequences. *Firmicutes* plays a vital role in the degradation of polysaccharides and oligosaccharides (39), which involves some key metabolic conversions by the gut microbial community (40). In addition, maternal synbiotic supplementation increased the abundances of *Butyricicoccus* and *Sharpea* belonged to *Firmicutes*. *Butyricicoccus* can reduce the production of pro-inflammatory cytokines to inhibit the host's inflammation (41). We found that maternal synbiotic supplementation increased *Butyricicoccus* abundance, which might reduce the inflammation occurrence of suckling piglets *via* altering gut microbiota composition and abundance. *Sharpea* promotes SCFAs (especially butyrate) and lactate production (42). Our study showed that maternal synbiotic supplementation increased *Sharpea* abundance in the offspring, which may favor inhibition of the proliferation of potential pathogenic bacteria by reducing the gut pH value. Additively, *Fusobacterium* can use glucose as a carbon source, the abundance of which is increased by polysaccharide degradation (43). Several studies reported that *Fusobacterium* might be a contributing factor for inflammation (44), the abundance of which increased in neonatal piglets with diarrhea (45). In the present study, the *Fusobacterium* abundance showed a decreased trend in the synbiotic group, implying that maternal synbiotic supplementation reduced this potential pathogenic bacteria.

Colonic SCFAs can exert crucial effects on intestinal function and health of the host before and after absorption in the blood (46). In addition of providing 60–70% of total energy to colonic cells (47), the SCFAs are associated with the reduction of the host's inflammation (48) and the relieving symptoms of other metabolic diseases (49). Among them, propionate reduces the serum cholesterol level and liver lipogenesis of rats (50). Our study showed that maternal synbiotic supplementation decreased propionate level in the colonic content. These findings suggested that maternal synbiotic supplementation increased certain gut microbiota species and promoted the production of specific metabolites. In addition, colonic *p_Firmicutes;g_Anaerostipes* abundance was positively correlated with acetate and SCFAs levels; and *Fusobacteria* and *p_Fusobacteria;g_Fusobacterium* abundances were negatively correlated with acetate level, suggesting that *Anaerostipes* might promote the SCFAs production while *Fusobacteria* and *Fusobacterium* would diminish them by a underlying mechanism that needs to be determined.

Cytokines can regulate the systemic inflammatory response of the body. The SCFAs promote the migration of leukocytes to the inflammatory site and production of several anti- and pro-inflammatory cytokines, including TNF-α, IL-1β, IL-2, IL-6, and IL-10 (51). Acetate, propionate, and butyrate reduce the production of TNF-α (52), IL-1β, and IL-6 (53). Interestingly, we found that maternal synbiotic supplementation decreased the plasma levels of TNF-α, IL-1β, IL-2, and IL-6 in offspring piglets, suggesting that dietary synbiotics might reduce inflammation in piglets via modifying several bacterial metabolite productions. Additionally, cytokines have the function of regulating immune and inflammatory responses and maintaining barrier integrity (54). In the present study, maternal synbiotics up-regulated the mRNA expression of colonic mucosal IFN-α, suggesting that the synbiotic addition in the maternal diets enhances the immune response of suckling piglets via regulating gut microbiota composition and metabolic activity as previously proposed (55).

The SCFAs can modulate hormone secretion (e.g., Leptin) (56) and are involved in modulating the production of Ghrelin (57). CCK can suppress the appetite by acting on the central nervous system (58). Ghrelin can act on appetite (59) and satiety by regulating the gut microbial community of the host. The PP secretion can be stimulated by dietary fat (60). Our study showed that maternal synbiotic supplementation decreased the plasma levels of Ghrelin, CCK, and PP of piglets, suggesting that maternal synbiotic addition might affect plasma hormone secretion of suckling piglet by mediating gut microbiota and their metabolites.

When the intestinal mucosal barrier is damaged, the permeability of which would increase, thus causing intestinal inflammation or other diseases due to harmful substances invading the body tissues (55). Compared with the antibiotic group, dietary synbiotic supplementation up-regulated the mRNA expression of colonic mucosal E-Cadherin, Occludin, ZO-1, and ZO-2, suggesting that the maternal synbiotic administration might improve tight-junction integrity of colonic intestinal epithelial cells via colonic microbiota. Shi et al. (61) found that the mixture of Lactobacillus species increased the colonic mucosal tight-junction proteins and relieved inflammation in antibiotic-supplemented mice by modulating their microbiota structure. Yin et al. (62) also showed that dietary XOS supplementation improved the intestinal barrier by up-regulating ZO-1 expression. Further work is required to explore the dose of synbiotic supplementation in maternal diets presenting an impact on the intestinal permeability in piglets.

In conclusion, maternal synbiotic supplementation from pregnancy to lactation may improve glycolipid and protein metabolism, reduce oxidative stress level, and improve the intestinal health of suckling piglets. Notably, these findings provide a new perspective for manipulating gut microbiota with synbiotic addition to improve the nutrient metabolism and intestine health of offspring. The changes in maternal milk composition after maternal synbiotic supplementation need further analysis in the future to full interpret the findings of the present study.

## Data Availability Statement

The datasets presented in this study can be found in online repositories. The names of the repository/repositories and accession number(s) can be found below: https://www.ncbi.nlm.nih.gov/, PRJNA609410.

## Ethics Statement

The animal study was reviewed and approved by Animal Care and Use Committee of the Institute of Subtropical Agriculture. Written informed consent was obtained from the owners for the participation of their animals in this study.

## Author Contributions

XK designed the experiment. CM, QG, WZ, QZ, HD, and WT carried out the animal trail, and sample collection and analysis. CM and WZ performed the statistical analyses. CM wrote the manuscript. FB and XK revised the manuscript. All authors reviewed this manuscript.

## Conflict of Interest

The authors declare that the research was conducted in the absence of any commercial or financial relationships that could be construed as a potential conflict of interest.
