# Supplementary material for: Supplementing Synbiotic in Sows' Diets Modifies Beneficially Blood Parameters and Colonic Microbiota Composition and Metabolic Activity in Suckling Piglets
Source: Front Vet Sci. 2020 Nov 30;7:575685. doi: 10.3389/fvets.2020.575685 (PMC7734190; doi:10.3389/fvets.2020.575685)
Supplement: Supplementary file 1 [file Data_Sheet_1.docx]

Supplementing synbiotics in sows’ diets modifies beneficially in piglet offspring blood parameters, and colonic microbiota composition and metabolic activity

Cui Ma^1, 2^, Qiankun Gao^1^, Wanghong Zhang^1, 2^, Qian Zhu^1, 2^, Wu Tang^1, 2^, Francois Blachier^3^, Hao Ding^1^, Xiangfeng Kong^1*^

^1^ CAS Key Laboratory of Agro-ecological Processes in Subtropical Region, Hunan Provincial Key Laboratory of Animal Nutritional Physiology and Metabolic Process, National Engineering Laboratory for Pollution Control and Waste Utilization in Livestock and Poultry Production, Institute of Subtropical Agriculture, Chinese Academy of Sciences, Changsha, Hunan 410125, China

^2^ University of Chinese Academy of Sciences, Beijing 101408, China

^3^ Université Paris-Saclay, AgroParisTech, INRAE, UMR PNCA, Paris 75005, France

^*^Correspondence:

Xiangfeng Kong

[nnkxf@isa.ac.cn](mailto:nnkxf@isa.ac.cn)

**Supplementary Table 1** Composition and nutrient levels of the basal diets (air-dry basis; %)

| Items | Pregnant sows' diet | Lactating sows' diet |
| --- | --- | --- |
| Ingredients |  |  |
| Corn | 37.50 | 66.00 |
| Soybean meal | 9.50 | 25.00 |
| Wheat bran | 14.00 | 5.00 |
| Barley | 25.00 |  |
| Soybean hull | 10.00 |  |
| Pregnant sows’ premix^1)^ | 4.00 |  |
| Lactating sows’ premix^2)^ |  | 4.00 |
| Total | 100.00 | 100.00 |
| Nutrient levels^3)^ |  |  |
| Digestible energy (MJ/Kg) | 12.55 | 13.87 |
| Crude fiber | 4.56 | 2.87 |
| Crude fat | 2.74 | 2.99 |
| Crude protein | 12.82 | 16.30 |
| SID Lys | 0.48 | 0.75 |
| SID Met+ Cys | 0.43 | 0.51 |
| SID Thr | 0.37 | 0.53 |
| SID Trp | 0.13 | 0.17 |
| Calcium | 0.62 | 0.65 |
| Phosphorus | 0.47 | 0.50 |

^1)^ Pregnant sows’ premix provided the following per kg of diets: CaHPO_4_⋅2H_2_O 10 g, NaCl 4 g, CuSO_4_⋅5H_2_O 80 mg, FeSO_4_⋅H_2_O 360 mg, ZnSO_4_⋅H_2_O 240 mg, MnSO_4_⋅H_2_O 100 mg, MgSO_4_⋅7H_2_O 1 g, 1% ICl 50 mg, 1% Na_2_SeO_3_ 36 mg, 1% CoCl₂16 mg, NaHCO_3_ 1.4 g, VA 10000 IU, VD_3_ 1800 IU, VE 20 mg, VK_3_ 2.4 mg, VB_1_ 1.6 mg, VB_2_ 6 mg, VB_6_ 1.6 mg, VB_12_ 0.024 mg, folic acid 1.2 mg, nicotinamide 20 mg, pantothenic acid 12 mg, biotin 0.12 mg, ferrous glycinate 100 mg, choline chloride 1 g, phytase 200 mg, fruity 80 mg, limestone 12 g.

^2)^ The lactating sows’ premix provides the following per kilogram of the diet: CaHPO_4_⋅2H_2_O 10 g, NaCl 4 g, CuSO_4_⋅5H_2_O 80 mg, FeSO_4_⋅H_2_O 360 mg, ZnSO_4_⋅H_2_O 240 mg, MnSO_4_⋅H_2_O 100 mg, 1% ICl 50 mg, 1% Na_2_SeO_3_ 36 mg, 1% CoCl₂16 m, NaHCO_3_ 1.4 g, VA 10000 IU, VD_3_ 1800 IU, VE 20 mg, VK_3_ 2.4 mg, VB_1_ 1.6 mg, VB_2_ 6 mg, VB_6_ 1.6 mg, VB_12_ 0.024 mg, folic acid 1.2 mg, nicotinamide 20 mg, pantothenic acid 12 mg, biotin 0.12 mg, 70% Lysine 1.5g, ferrous glycinate 100 mg, choline chloride 1 g, phytase 200 mg, fruity 80 mg, limestone 12 g.

^3)^ Nutrient levels were calculated values.

SID, standard ileum digestible.

**Supplementary Table 2** Primer sequences determined in this study

| Items | Primer sequence (5'-3') | Accession number | Size (bp) |
| --- | --- | --- | --- |
| Actin | F: GATCTGGCACCACACCTTCTACAAC  R：TCATCTTCTCACGGTTGGCTTTGG | XM 021086047.1 | 107 |
| E-cadherin | F: GAAGGAGGTGGAGAAGAGGAC  R: AGAGTCATAAGGTGGGGCAGT | NM 001163060.1 | 119 |
| IFN-α | F: ATCTGCTCTCTGGGCTGTGACC  R: CTCATTTGTGCCAGGAGCCTCAG | NM 001195375.1 | 80 |
| IL-1β | F: AAGAGGGACATGGAGAAGCGATTTG  R: TTGTTCTGCTTGAGAGGTGCTGATG | XM 021085847.1 | 114 |
| IL-2 | F: TGCACTAACCCTTGCACTCA  R: CAACTGTAAATCCAGCAGCAA | XM021100436.1 | 100 |
| IL-10 | F: GGGCTATTTGTCCTGACTGC  R: GGGCTCCCTAGTTTCTCTTCC | NM 214041.1 | 105 |
| Occludin | F: CAGTGGTAACTTGGAGGCGTCTTC  R: CGTGTAGTCTGTCTCGTAATGGTCTTG | NM 001163647.1 | 100 |
| TNF-α | F: GCACTGAGAGCATGATCCGAGAC  R: CGACCAGGAGGAAGGAGAAGAGG | NM 214022.1 | 120 |
| ZO-1 | F: TCCTGAGTTTGATAGTGGCGTTGAC  R: CACGGTGTGACCATCCTCATCTTC | AJ318101.1 | 148 |
| ZO-2 | F: CCCTCAGCCGTTGCCAGTAATG  R: GCCCTCTCCCACCTCGTCAC | NM 001206404.1 | 147 |

Note: IFN-α, interferon-α; IL-1β, interleukin-1β; IL-2, interleukin-2; IL-6, interleukin-6; IL-10, interleukin-10; TNF-α, tumor necrosis factor-α; ZO-1, Zonula Occluden-1; ZO-2, Zonula Occluden-2.

**
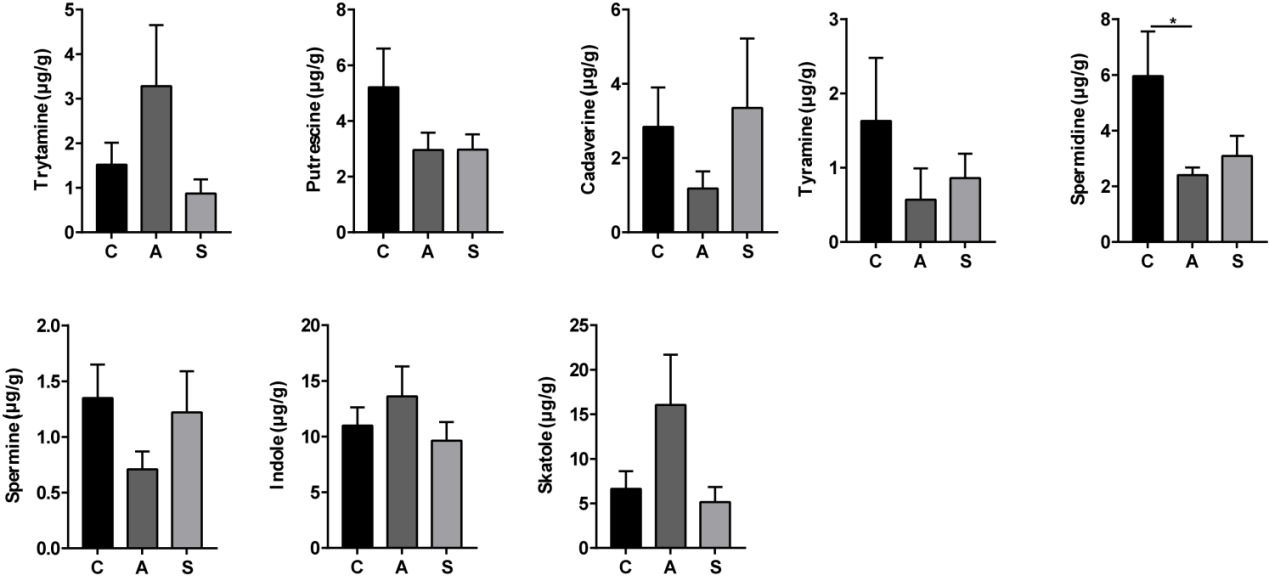
**

**Fig. S1 Effect of maternal synbiotic supplementation on colonic metabolite levels of suckling Bama mini-piglets.** C, A, and S present the control group, antibiotic group, and synbiotic group, respectively. The data were analyzed by Duncan’s multiple-range test using One-way analysis of variance. Data represent the means ± SEM. **P* < 0.05. *n*=8 per group.
